# Optical Forces on Chiral Particles: Science and Applications

**DOI:** 10.3390/mi15101267

**Published:** 2024-10-17

**Authors:** Weicheng Yi, Haiyang Huang, Chengxing Lai, Tao He, Zhanshan Wang, Xinhua Dai, Yuzhi Shi, Xinbin Cheng

**Affiliations:** 1 Institute of Precision Optical Engineering, School of Physics Science and Engineering, Tongji University, Shanghai 200092, China; 2311335@tongji.edu.cn (W.Y.); 1953799@tongji.edu.cn (H.H.); 1950621@tongji.edu.cn (C.L.); hetao@tongji.edu.cn (T.H.); wangzs@tongji.edu.cn (Z.W.); chengxb@tongji.edu.cn (X.C.); 2 MOE Key Laboratory of Advanced Micro-Structured Materials, Shanghai 200092, China; 3 Shanghai Institute of Intelligent Science and Technology, Tongji University, Shanghai 200092, China; 4 Shanghai Frontiers Science Center of Digital Optics, Shanghai 200092, China; 5 Technology Innovation Center of Mass Spectrometry for State Market Regulation, Center for Advanced Measurement Science, National Institute of Metrology, Beijing 100029, China

**Keywords:** optical force, chiral particle, optical manipulation, chirality

## Abstract

Chiral particles have attracted considerable attention due to their distinctive interactions with light, which enable a variety of cutting-edge applications. This review presents a comprehensive analysis of the optical forces acting on chiral particles, categorizing them into gradient force, radiation pressure, optical lateral force, pulling force, and optical force on coupled chiral particles. We thoroughly overview the fundamental physical mechanisms underlying these forces, supported by theoretical models and experimental evidence. Additionally, we discuss the practical implications of these optical forces, highlighting their potential applications in optical manipulation, particle sorting, chiral sensing, and detection. This review aims to offer a thorough understanding of the intricate interplay between chiral particles and optical forces, laying the groundwork for future advancements in nanotechnology and photonics.

## 1. Introduction

Light carries momentum [1], and its interaction with matter has long captivated the scientific community, prompting extensive research. Optical forces arise from the transfer of momentum between photons and particles, a phenomenon governed by the principles of electrodynamics. When light interacts with a particle, it can exert forces that facilitate applications such as optical trapping and manipulation. The pioneering work of Arthur Ashkin [2,3] in the 1970s, which culminated in the invention of optical tweezers, represents a pivotal breakthrough in this field. Ashkin demonstrated that highly focused laser beams could trap and manipulate small particles [3,4], enabling diverse applications in both biological and physical sciences. This groundbreaking discovery paved the way for investigating the specific interactions between light and various types of particles [5,6], including chiral particles.

Chirality is an asymmetrical property where an object cannot be superimposed onto its mirror image. This characteristic is ubiquitous in various entities, including amino acids, sugars, synthetic chiral molecules, and nanoparticles. Chiral particles [6,7,8,9] can uniquely interact with circularly polarized light to generate optical force related to the chirality of the particles, which is distinct from, and generally lesser than, the usual optical force acting on achiral particles. They exhibit intriguing behaviors, such as differential scattering and absorption of left- and right-circularly polarized light, a phenomenon known as circular dichroism. These interactions form the foundation for the distinct optical forces that can be harnessed for the precise manipulation and separation of chiral entities [4,10].

Optical forces acting on chiral particles [5,10,11,12,13,14,15,16,17,18] are influenced by various factors, including the particle’s size, shape, material properties, and the polarization state of the incident light. These forces can be broadly classified into optical gradient forces (OGFs), optical scattering forces (optical radiation pressure) [16], optical pulling forces (OPFs), optical lateral forces (OLFs), and optical forces on coupled chiral particles. A thorough understanding of these forces is crucial for their practical application. The OGF arises from the intensity gradient of the optical field, trapping particles in regions of high light intensity. For chiral particles, the gradient force is highly dependent on both the polarization state of the light and the particle’s chirality. The radiation pressure results from the momentum transfer between photons and particles. In chiral particles, this force can exhibit anisotropy, depending on the particle’s chirality and the light’s polarization. Under certain conditions, chiral particles may experience a pulling force that opposes the direction of light propagation—a counterintuitive phenomenon with significant implications for optical manipulation and transport. Chiral particles can also experience transverse forces that act perpendicular to the direction of light propagation, due to interactions with the light’s helicity. OLF is particularly intriguing for applications in directing and sorting particles. When multiple chiral particles interact with each other or with structured light fields, optical forces on coupled chiral particles can emerge, leading to complex dynamics and potential applications in self-assembly and collective behavior.

**Figure 1:**
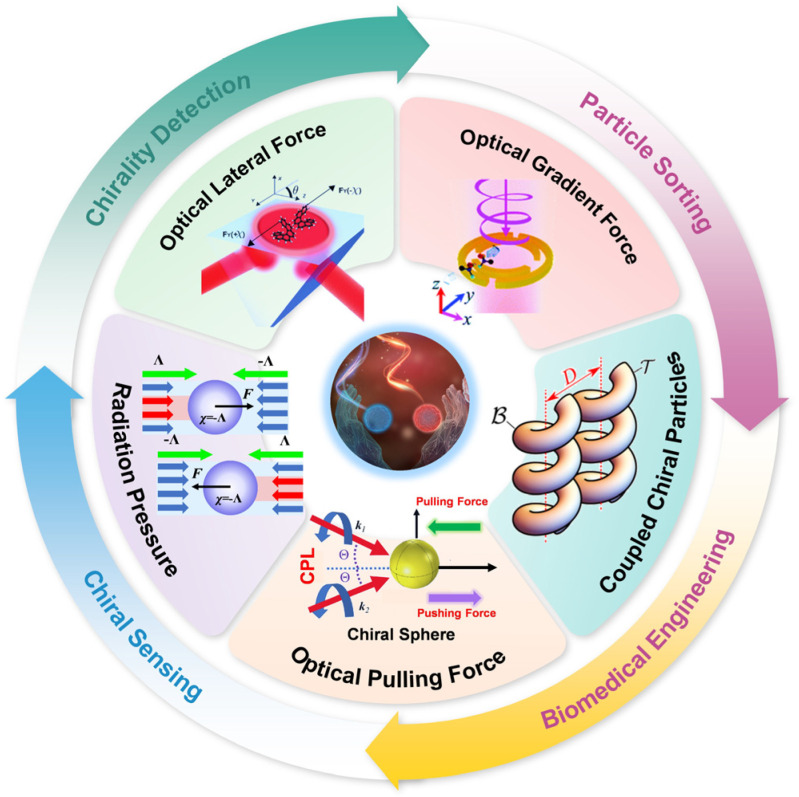
Schematic illustration of different types of optical forces on chiral particles and applications. Center of the schematic illustration, reproduced with permission from ref. [23]. Optical gradient force, reproduced with permission from ref. [24]. Copyright @2019 the Owner Societies. Radiation pressure, reproduced with permission from ref. [25]. Copyright @2013 American Physical Society. Optical pulling force, reproduced with permission from ref. [26]. Copyright @2021 Optical Society of America. Optical lateral force, reproduced with permission from ref. [27]. Copyright @2015 Proceedings of the National Academy of Sciences. Coupled chiral particles, reproduced with permission from ref. [28]. Copyright @2020 American Physical Society.

This review investigates the physical mechanisms underlying the optical forces acting on chiral particles, as illustrated in Figure 1. This discussion is organized into the following categories: OGF, optical radiation pressure, OPF, OLF, and optical forces on coupled chiral particles. Additionally, we will briefly examine the practical applications of these optical forces, including the precise trapping and manipulation [4,12,19] of chiral particles, enhanced detection of molecular chirality [20,21], and techniques for the efficient separation of chiral entities [14,22]. This review analyzes these mechanisms and applications to provide a comprehensive insight into the principles and potential uses of optical forces on chiral particles, paving the way for future innovations in nanotechnology and photonics.

## 2. Physical Mechanics

The constitutive relation of chiral particles is well established and is given by [29,30,31]:

(1) $$\begin{array}{l} D=\varepsilon_{p}E+i\kappa \sqrt{\varepsilon_{0}\mu_{0}}H,\\ B=-i\kappa \sqrt{\varepsilon_{0}\mu_{0}}E+\mu_{p}H, \end{array}$$

where $\varepsilon_{p}$ and $\mu_{p}$ are permittivity and permeability of the particle, respectively, and κ is the chirality parameter, constrained by the inequality $\kappa <\sqrt{\varepsilon \mu },$ ε and μ are relative permittivity and permeability of the medium [32,33].

The induced electromagnetic dipole moments in chiral particles are determined by the incident electromagnetic field and the corresponding polarizabilities [34,35,36,37]:

(2) $$\begin{array}{l} d=\alpha_{ee}E+\alpha_{em}B,\\ m=-\alpha_{em}E+\alpha_{mm}B, \end{array}$$

where E and B represent the incident electric and magnetic fields, respectively, $\alpha_{ee}$, $\alpha_{mm}$, and $\alpha_{em}$ are the polarizabilities of the particles. The polarizability $\alpha_{em}$ is related to the chirality parameter κ of the particles and varies with the sign of κ. For achiral particles, $\alpha_{em}=0$. The polarizabilities $\alpha_{ee}$, $\alpha_{mm}$, and $\alpha_{em}$ are related to the Mie coefficients $a_{1}$, $b_{1}$, and $c_{1}$:

(3) $$\alpha_{ee}=\frac{i6\pi \varepsilon \varepsilon_{0}}{k^{3}}a_{1},\;\alpha_{mm}=\frac{i6\pi \mu \mu_{0}}{k^{3}}b_{1},\;\alpha_{em}=\frac{6\pi }{\omega k^{2}}c_{1}.$$

The scattering coefficients are defined as follows:

(4) $$a_{n}=\frac{V_{n}(R)A_{n}(L)+V_{n}(L)A_{n}(R)}{W_{n}(L)V_{n}(R)+W_{n}(R)V_{n}(L)},$$

(5) $$b_{n}=\frac{W_{n}(L)B_{n}(R)+W_{n}(R)B_{n}(L)}{W_{n}(L)V_{n}(R)+W_{n}(R)V_{n}(L)},$$

(6) $$c_{n}=i\frac{W_{n}(R)A_{n}(L)-W_{n}(L)A_{n}(R)}{W_{n}(L)V_{n}(R)+W_{n}(R)V_{n}(L)},$$

with

(7) $$W_{n}(J)=m\psi_{n}\left(m_{J}X\right){\xi^{\prime }}_{n}(X)-\xi_{n}(X){\psi^{\prime }}_{n}\left(m_{J}X\right),$$

(8) $$V_{n}(J)=\psi_{n}\left(m_{J}X\right){\xi^{\prime }}_{n}(X)-m\xi_{n}(X){\psi^{\prime }}_{n}\left(m_{J}X\right),$$

(9) $$A_{n}(J)=m\psi_{n}\left(m_{J}X\right){\psi^{\prime }}_{n}(X)-\psi_{n}(X){\psi^{\prime }}_{n}\left(m_{J}X\right),$$

(10) $$B_{n}(J)=\psi_{n}\left(m_{J}X\right){\psi^{\prime }}_{n}(X)-m\psi_{n}(X){\psi^{\prime }}_{n}\left(m_{J}X\right).$$

Here, X=ka, a represents the particle’s radius and J=L,R stands for left and right chirality, respectively; $\psi_{n}\left(\rho \right)=\rho j_{n}\left(\rho \right)$, $\xi_{n}\left(\rho \right)=\rho h_{n}^{\left(1\right)}\left(\rho \right)$, $j_{n}\left(\rho \right)$, and $h_{n}^{\left(1\right)}\left(\rho \right)$ denote the spherical Bessel function and the first kind of spherical Hankel function; $\psi_{n}\prime \left(\rho \right)$ and $\xi_{n}\prime \left(\rho \right)$ represent the differentiation of $\psi_{n}\left(\rho \right)$ and $\xi_{n}\left(\rho \right)$, respectively. The relative refractive indices $m_{L}$, $m_{R}$ and the averaged refractive index m can be expressed as $m_{L}=\sqrt{\varepsilon_{p}\mu_{p}/\varepsilon \mu }+\kappa$, $m_{R}=\sqrt{\varepsilon_{p}\mu_{p}/\varepsilon \mu }-\kappa$, and $m=(m_{L}+m_{R})/2$, respectively.

The time-averaged optical force exerted by the electromagnetic field **E** and **B** on a dipolar particle is given by [6,32,38]:

(11) F=12Re{d⋅(∇)E*+m⋅(∇)B*−k46πεc(d×m*)}.

From these equations, the expression for the optical force on chiral particles can be derived as follows [35,39]:

(12) 〈F〉=Fgrad+Frad+Fcurl+Fspin+Fflow=−∇〈U〉+σ〈P〉+[σe∇×Se+σm∇×Sm−Imαem∇×〈P〉]+[γeω2Se+γmω2Sm]+ωk312πImαeeαmm*−Imαemαem*ImE×H*,

Here, $F_{\mathrm{grad}}$ denotes the OGF, with its potential given by U=−14ReαeeE2−14ReαmmH2−12ReαemImE⋅H*; $F_{\mathrm{rad}}$ is the optical radiation pressure, with σ=σec2+σmc2−ωk36πReαeeαmm*+Reαemαem* being the extinction cross-section [40]; the force $F_{\mathrm{curl}}$ originates from $\nabla \times \langle P\rangle$ (P is the Poynting vector); $F_{\mathrm{spin}}$ results from the contributions of $S_{e}$ and $S_{m}$, with $S_{e}$ and $S_{m}$ representing the electric and magnetic spin angular momentum (SAM), respectively. γe=−2εμImαem+k3μ3πε0Reαeeαem* and γm=−2εμImαem+k3ε3πμ0Reαemαmm*; and $F_{\mathrm{flow}}$ is associated with the imaginary component of the Poynting vector.

The optical forces on chiral particles can be categorized into five components based on their origins. To elucidate the specific effects of each force on chiral particles, the subsequent sections will classify the outcomes produced by these optical forces into five categories: OGF, optical radiation pressure, OPF, OLF, and the optical forces on coupled chiral particles. We will introduce each force’s definition, mechanism, and dynamic effects on chiral particles in detail.

### 2.1. Optical Gradient Force on Chiral Particles

The OGF arises from the inhomogeneous intensity of the optical field and is directed along the gradient of light intensity. This force either attracts particles toward regions of higher intensity or repels them from such regions. OGF is fundamental to the operation of optical tweezers, which have a wide range of applications including optical trapping, bioprospecting, particle sorting, biomolecule stretching, and the study of interactions between biological entities. Specifically, chiral OGF refers to the component of the gradient force that is influenced by the chirality of the particles. Unlike conventional OGF components, which depend on the intensity inhomogeneity of the incident light field, chiral OGF can be generated even in an optical field with uniform light intensity [41].

**Figure 2:**
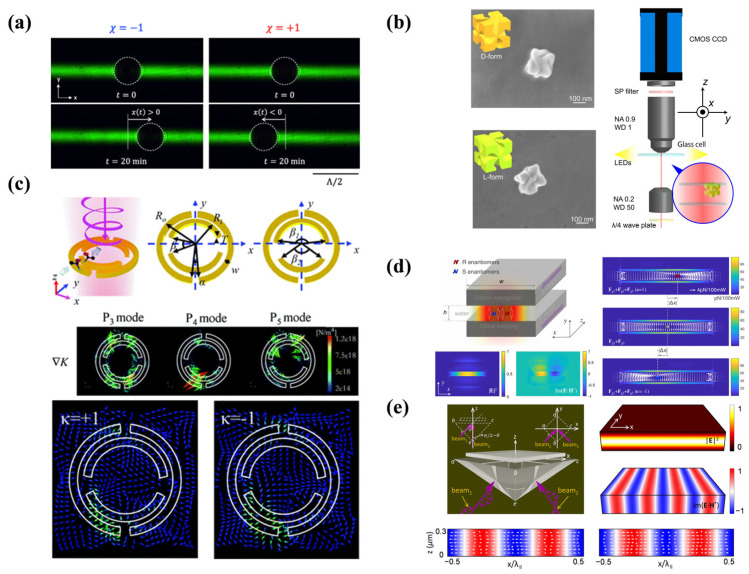
OGF on chiral particles. (**a**) Optical Stern–Gerlach Newton experiment using chiral liquid crystal microsphere, reproduced with permission from ref. [42]. Copyright @2019 American Physical Society. (**b**) Scanning electron microscope image of chiral gold nanoparticles and schematic diagram of chiral gold nanoparticle capture experiment, reproduced with permission from ref. [43]. Copyright @2022 American Association for the Advancement of Science. (**c**) The chiral gradient force acting on chiral nanoparticles that can overcome non-chiral force when specimens are placed in a Fano-enhanced chiral gradient near-field, reproduced with permission from ref. [24]. Copyright @2019 the Owner Societies. (**d**) Optical trapping separation in the counterpropagating slot waveguides, reproduced with permission from ref. [45]. Copyright @2021 American Physical Society. (**e**) The enantiomer sorting through the light field generated by the two-wave superposition within the gap between the top and bottom substrates, reproduced with permission from ref. [50]. Copyright @2024 American Institute of Physics.

Experimentally, Kravets et al. [42] generated a light field with a one-dimensional gradient of chiral density by interfering two slightly non-collinear laser beams with orthogonal linear polarization states, as illustrated in Figure 2a. They designed a chiral optical Stern–Gerlach sorter to sort chiral liquid crystal microspheres, where chiral liquid crystal microspheres are selectively displaced by means of optical forces arising from optical helicity gradients. As depicted in Figure 2b, Yamanishi et al. [43] observed chiral gradient forces acting on chiral gold nanoparticles, which depended on both the handedness of the circularly polarized light and the chirality of the particles. This breakthrough introduces a novel optical method for manipulating and separating chiral materials using circularly polarized light.

In general, Re(αem) is much smaller than Re(αee) for small particle materials, resulting in the chiral OGF being much smaller than other non-chiral components, which impedes enantiomer sorting. Therefore, enhancing the chiral OGF remains an important area of research.

Plasmonic structures can significantly amplify the optical field gradient, thereby enhancing the chiral OGF. Zhao et al. [44] achieved enantiomer sorting using coaxial plasmonic apertures. Under circularly polarized light, opposite enantiomers experience distinct trapping forces due to the OGF: one is captured in a deep potential well, while the other is repelled by a potential barrier. As shown in Figure 2c, Cao et al. [24] employed plasmonic nanoapertures to generate a Fano-enhanced chiral gradient near-field, which further amplified the chiral OGF. Additionally, Fang et al. [45] designed a subwavelength silicon-based waveguide platform, as illustrated in Figure 2d, offering a novel approach to enhance the chiral OGF. The strongly confined standing evanescent fields within the gap of slot waveguides can significantly amplify the gradient force, enabling nanoscale enantiomer separation.

Beyond these methods, the chiral OGF can also be significantly enhanced using superchiral fields [46,47], core–shell structures [48], and photonic crystals [49]. However, most of these current approaches simultaneously enhance both chiral and achiral optical forces. Yao et al. [50] utilized a homogeneous light field, generated by the interference of two waves, to achieve enantiomeric separation, as shown in Figure 2e. This experimental setup eliminates the achiral optical gradient force, allowing for the selective manipulation of chiral particles. The figure depicts the light field between two substrates, where enantiomers experience different optical forces, leading to their separation.

### 2.2. Optical Radiation Pressure on Chiral Particles

Light carries both linear and angular momentum, which can be transferred to particles, enabling interactions with matter and facilitating optical manipulation. Optical radiation pressure, the mechanical effect of linear momentum transfer from light to matter, occurs when light is refracted, reflected, scattered, or absorbed. This phenomenon is prominently applied in atomic refrigeration, optical sorting, particle propulsion, and other specialized areas.

A notable example of light’s linear momentum transfer is the generation of radiation pressure, which propels solar sails in the direction of light wave propagation. The 1980s saw significant advancements in optical trapping and the study of radiation pressure, culminating in the 1997 Nobel Prize in Physics awarded to Steven Chu, Claude Cohen-Tannoudji, and William D. Phillips for their pioneering work in trapping and cooling atoms using radiation pressure [51,52,53,54,55,56].

**Figure 3:**
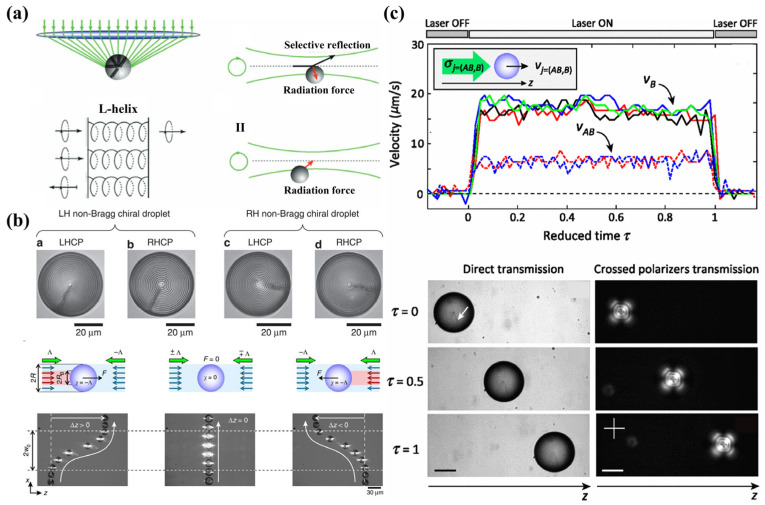
Optical radiation pressure on chiral particles. (**a**) Optical trapping and repulsion of a radial isotropic microsphere, reproduced with permission from ref. [57]. Copyright @2011 John Wiley & Sons, Inc. (**b**) Optical sorting of droplets with different chirality, reproduced with permission from ref. [58]. Copyright @2014 Springer Nature. (**c**) Motion of spherical radial cholesteric droplet under irradiation of a single beam, reproduced with permission from ref. [25]. Copyright @2013 American Physical Society.

In 2008, B.M. Ross and A. Lakhtakia explored the circular Bragg reflection mechanism in cholesteric systems under the framework of radiation pressure. Their study leveraged chiral sculptured thin films and demonstrated that the radiation pressure on these “chiral mirrors” is influenced by the handedness of both the incident light and the mirror itself [16]. Further insights were gained from the experimental work of G. Cipparrone et al. [57] in 2011, who observed a chiral optical force. This research involved trapping micron-sized chiral cholesteric beads in both stable and unstable optical potentials. The forces examined were chiral, derived from the beads’ chiroptical properties as circular Bragg reflectors [57], as illustrated in Figure 3a. These reflectors selectively reflect or transmit left- or right-circularly polarized light, creating a force equilibrium in the optical trap dependent on the helicity of the trapping beam. This work explored the pressure exerted by circularly polarized plane waves on large chiral spherical or other configured particles and analyzed the transfer of angular momentum between light and chiral substances. This understanding has been extensively applied to the sorting of chiral cholesteric microdroplets [12,13,25,57,58], as depicted in Figure 3b,c.

To more accurately calculate the radiation force and torque exerted on particles by a laser beam, researchers have developed several theoretical models and methodologies. In addition to numerical approaches such as the T-matrix method [59], moment method [60,61], finite-difference time-domain (FDTD) method [62], and finite-difference frequency-domain (FDFD) method [63], analytical methods offer precise solutions, allowing for a thorough investigation of the scattering properties of chiral particles. Gordon was the first to derive the scattered amplitude matrix for chiral particles using the Generalized Lorenz–Mie Theory (GLMT) [64]. Shang et al. calculated the radiation force and torque on a chiral sphere illuminated by a Gaussian beam within the GLMT framework, discussing the effects of the chirality parameter on the radiation force and torque [65].

Wu et al. later proposed an analytical method to analyze the scattering of large chiral particles and derived the corresponding scattering coefficients. Recently, Cui et al. numerically investigated the light scattering of Laguerre–Gaussian (LG) vortex beams and developed analytical solutions for the interaction of LG beams with chiral particles of arbitrary shapes. They derived the electric and magnetic field components of incident LG vortex beams using the vector potential method [66]. In 2020, Zheng et al. developed general formulations for computing optical gradient and scattering forces on spherical chiral particles immersed in generic monochromatic optical fields [67]. These comprehensive and rigorous theories and methods have significantly enhanced our understanding of chiral optical manipulation, playing a crucial role in the sorting of chiral particles and the detection of chirality.

### 2.3. Optical Pulling Force on Chiral Particles

Previous studies have successfully demonstrated optical manipulation techniques, such as pushing, trapping, and rotating tiny objects ranging from nanometers to tens of micrometers in size. However, manipulating objects through backward pulling has remained elusive and counterintuitive. Generally, with a standard beam lacking a longitudinal gradient, it is expected that an object illuminated by light would be pushed forward. Yet, about a decade ago, researchers discovered a new and counterintuitive phenomenon known as OPF, which pulls an object toward the light source [40,68,69,70]. Marston first predicted this intriguing phenomenon in acoustics in 2006 [71] and later extended it to optics [72]. This remarkable discovery has sparked significant interest in both fundamental physics and potential advanced manipulation applications. In this discussion, we primarily focus on the non-trivial OPF exerted on chiral objects.

The chirality of an object can be generally categorized into two distinct aspects: structural chirality and material chirality. These chiral properties affect how objects respond to left- and right-circularly polarized beams. Circularly polarized beams carry SAM, which can couple with the mechanical momentum of the object, offering a novel mechanism for realizing OPF [37].

**Figure 4:**
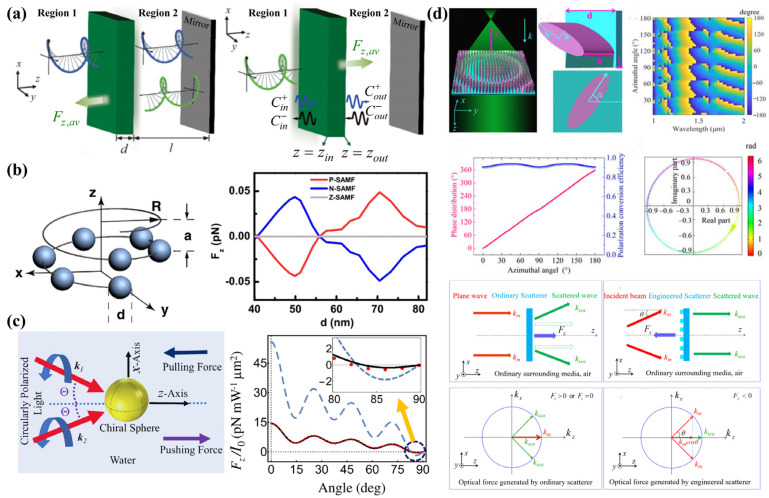
OPF on chiral particles. (**a**) A system with a flat mirror can flexibly adjust the sign of optical force on the chiral medium, reproduced with permission from ref. [73]. Copyright @2015 American Physical Society. (**b**) The schematic structure of the chiral chain, where the sign of optical force depends on the size of the structure, reproduced with permission from ref. [37]. Copyright @2014 American Physical Society. (**c**) Schematic diagram of a particle illuminated by two non-collinear circularly polarized laser beams, reproduced with permission from ref. [26]. Copyright @2010 Optical Society of America. (**d**) The mechanisms of OPF generation and the design process of the metalens photonic probe, reproduced with permission from ref. [77]. Copyright @2023 MDPI.

In 2015, Fernandes et al. [73] proposed a novel scheme for generating the OPF using chirality, as illustrated in Figure 4a. Intriguingly, they discovered that the optical force could be switched from positive to negative when the chirality of light is altered. It is well understood that a chiral slab exhibits different responses to left- and right-circularly polarized light. The transmission and reflection coefficients for these two polarizations are given as follows [74]:

(13) $$\begin{array}{l} T_{R,L}=\frac{4ze^{ik_{R,L}}d}{{\left(1+z\right)}^{2}-{\left(1-z\right)}^{2}e^{2ink_{0}d}},\\ R_{R,L}=\frac{{\left(1-z\right)}^{2}(e^{2ink_{0}d}-1)}{{\left(1+z\right)}^{2}-{\left(1-z\right)}^{2}e^{2ink_{0}d}}, \end{array}$$

where *d* represents the thickness of the chiral slabs, $k_{R,L}=k_{0}(n\pm \chi )$ is the wave vector, and $k_{0}=\omega /c,n=\sqrt{\mu /\varepsilon }$ is the normalized impedance. Analyses of the transmission and reflection coefficients reveal that, for those two kinds of chiral lights, the reflection coefficients are the same, while the transmission coefficients are dependent on polarization. In a system with an opaque mirror that reverses the handedness of the beam, the chirality of the incident light can be reversed by the mirror. Consequently, the chiral slab will be pulled toward the light source due to absorption. Alternatively, if the handedness of the incident beam is reversed, the slab will be pushed directly away. This study demonstrates the possibility for achieving an optical conveyor belt for chiral objects.

Meanwhile, Ding et al. [37] investigated the OPF associated with structural chirality. In this system, researchers created a chiral structure composed of metallic spheres (i.e., gold with $\varepsilon_{g}=-5+0.13i$ at the wavelength λ=337nm) arranged in a left-handed spiral, as shown in Figure 4b. Under the dipole approximation, for a plane wave propagating along the *z* direction, the optical force on the object in the *z* direction is

(14) Fz=14Re{Tee:α→ee+Tmm:α→mm}−k412πε0cRe{L~+S~}z.

where $T^{ee}$ and $T^{mm}$ are rank-3 tensors that depend only on the field. $\tilde{L}$ and $\tilde{S}$ are two auxiliary quantities that satisfy the following equations.

$$\tilde{L}=E\cdot {\left({\overset{↔}{\alpha }}^{em*}\times {\overset{↔}{\alpha }}^{ee}\right)}^{T}\cdot E^{*}-B\cdot {\left({\overset{↔}{\alpha }}^{mm†}\times {\overset{↔}{\alpha }}^{em}\right)}^{T}\cdot B^{*},$$

$$\tilde{S}=B\cdot {\left({\overset{↔}{\alpha }}^{em*}\times {\overset{↔}{\alpha }}^{em}\right)}^{T}\cdot E^{*}-E\cdot {\left({\overset{↔}{\alpha }}^{mm†}\times {\overset{↔}{\alpha }}^{ee}\right)}^{T}\cdot B^{*}.$$

From this equation, it is evident that the first term and $\tilde{S}$ are proportional to the linear momentum of the incident light, which is not dependent on the chirality of the object. However, the sign of $\tilde{L}$ changes with the chirality of the incident light, regardless of the direction of the incident light. These characteristics enable the generation of a negative pulling force. To isolate the chirality-dependent terms of the force, the researchers proposed using two incoherent plane waves (left- and right-circularly polarized) propagating in opposite directions. For different sizes of the spiral structure, the positive spin angular momentum flux (P-SAMF) can induce both positive and negative force, while the negative spin angular momentum flux (N-SAMF) generates a force opposite to that produced by P-SAMF, as shown in Figure 4b.

Recent studies have highlighted the novel degree of freedom provided by OPF, prompting extensive exploration into the relationship between OPF and object chirality. This research has potential applications across material science, chemistry, biology, and particularly the pharmaceutical industry, where many drugs are composed of single enantiomers [75]. Zheng et al. [76] proposed a straightforward approach for chirality sorting using OPF. They constructed an optical lattice with three plane waves of identical circular polarization. In this lattice, particles with a specific chirality are attracted to the periodic intensity maxima by the transverse optical force, where they are stably trapped and experience a pulling force along the negative z-axis. In contrast, particles with opposite chirality are repelled and move away from the intensity maxima, eventually dispersing into regions where they experience a positive (pushing) force along the z-axis. Meanwhile, Rfaqat Ali et al. [26] introduced a novel twofold scheme for all-optical enantioselection and sorting of multipolar chiral microspheres. This method utilizes two non-collinear, non-structured, circularly polarized light sources, as depicted in Figure 4c. Recently, the concept of using conventional lenses or prisms as photonic probes for OPF has been proposed, though their sizes are generally too large for effective optical manipulation. To address this issue, Peng et al. [77] designed a chiral metalens as a photonic probe capable of generating a robust OPF. In their system, the longitudinal optical force on the metalens exhibited a circular dichroism response when illuminated by light with different circular polarization states. By switching the polarization of the incident light, the longitudinal optical force can be flexibly altered from a pulling force to a pushing force, as shown in Figure 4d.

### 2.4. Optical Lateral Force on Chiral Particles

The OLF, a unique force acting perpendicular to the direction of light propagation and independent of both intensity and phase gradients, has garnered significant attention due to its intriguing underlying physics and potential applications in enantiomer sorting. The generation of an OLF relies on breaking the symmetry of the system, allowing light to transfer lateral momentum to an object. Various mechanisms can produce OLFs, including the coupling of light with the object’s chirality [29,34,78,79], spin-orbit interaction [80,81,82], and surface plasmon polaritons (SPPs) [83,84,85].

**Figure 5:**
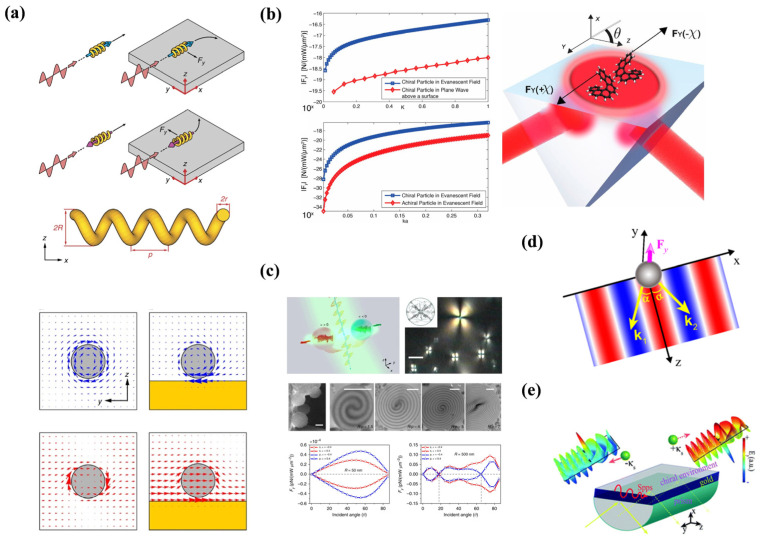
OLF on chiral particles. (**a**) OLF on chiral particles at interface resulted from coupling of chirality and linearly polarized light, reproduced with permission from ref. [34]. Copyright @2014 Springer Nature. (**b**) OLF on chiral particle in linearly polarized evanescent wave, reproduced with permission from ref. [27]. Copyright @2015 Proceedings of the National Academy of Sciences. (**c**) Reversible OLF generated by line-shaped linearly polarized beam, reproduced with permission from ref. [29]. Copyright @2020 Springer Nature. (**d**) Schematic diagram of OLF generated by chiral particle in dual-plane wave interference field, reproduced with permission from ref. [35]. Copyright @2016 American Physical Society. (**e**) Schematic diagram of metal–chiral Kretschmann configuration with SPP-assisted chiral enantiomer recognition and separation, reproduced with permission from ref. [83]. Copyright @2019 the Owner Societies.

When a chiral particle is placed at an interface and illuminated with linearly polarized light, the asymmetric coupling between the particle’s chirality and the interface can generate an OLF [34], as shown in Figure 5a. This OLF mainly originates from the asymmetric distribution of the Poynting momentum, namely $F_{\mathrm{rad}}$. Additionally, the spin density force resulting from substrate reflection also contributes to the generation of OLFs. Another approach for producing OLFs on chiral particles involves using linearly polarized light to generate evanescent waves. As illustrated in Figure 5b, Hayat et al. [27] employed linearly polarized waves to generate evanescent waves through total internal reflection, resulting in OLFs via the interaction between chirality and transverse SAM within the evanescent waves. In this scenario, there is no transverse field gradient or transverse wave propagation. OLFs arising from transverse SAM can also be observed in Bessel beams [86] and Laguerre–Gaussian beams [87]. Shi et al. [29] experimentally observed OLFs by directing linearly polarized light at chiral particles situated at an air–water interface, as shown in Figure 5c. The direction of the OLF is highly dependent on factors such as particle size, chirality, angle of incidence, and the polarization of the light beam. Additionally, Chen et al. [88] reported that OLFs can have opposite directions on pairs of particles with opposite chirality when illuminated by linearly polarized light.

Beyond manipulation with a single linearly polarized beam, an OLF can also arise from interfering waves or vortex beams. As depicted in Figure 5d, Chen et al. [35] demonstrated that two interfering plane waves can generate an OLF on chiral particles. The OLF can laterally push chiral nanoparticles depending on the particle’s chirality and the phase difference between the two waves. Zhang et al. [36] further investigated this phenomenon by exploring the optical force on chiral particles within an interference field formed by two plane waves with arbitrary polarization states. They found that an OLF can occur whether the plane waves are linearly or circularly polarized. Although the magnitude of the OLF varies with chirality and polarization, the direction of the particle’s motion remains consistent, complicating the separation of chiral particles. To address this sorting challenge, Shi et al. [29] immersed half of the nanoparticles in water and exposed the other half to air, enabling directional sorting of different chiral particles using linearly polarized light. More recently, Li et al. [89] proposed a mechanism for sorting chiral particles using a tightly focused vector beam, where the radial OLF in the vector beam can either capture or repel chiral particles, depending on their chiral parameters.

The OLF is generally weaker than the OGF and optical radiation force, which limits its applicability in a wide range of scenarios. Consequently, enhancing chiral-related OLFs has become a critical challenge. Alizadeh et al. [90] demonstrated that by spatially separating the maxima of the transverse spin density from the field intensity gradient, it is possible to prioritize the chiral-specific component of the force over the non-chiral component. They showed that chiral optical forces, which can surpass both gradient and scattering forces, can arise near optical nanofibers and nanowires. As illustrated in Figure 5e, Zhang et al. [83] generated a chiral SPP in a chiral environment, resulting in a chiral-sensitive, robust OLF capable of detecting and separating chiral particles. This OLF holds significant potential for enantiomeric sorting. This phenomenon could serve as a valuable tool for calculating enhanced optical forces on Mie chiral particles. Zhu et al. [78] explored the superposition effects of multipoles in single chiral structures and found that combining two toroidal dipoles or electromagnetic sextupoles can generate a hybrid mode that significantly amplifies the OLF to levels comparable to optical radiation pressure. Additionally, these modes can interfere destructively, which suppresses side scattering and thereby reduces the OLF.

### 2.5. Optical Force on Coupled Chiral Particles

When chiral particles are coupled, the interplay between their chiral properties and the optical forces they experience becomes increasingly complex and intriguing. Although the optical forces on a single chiral particle in the Rayleigh regime have been extensively studied, the behavior of optical forces in coupled chiral particles remains relatively unexplored. This section briefly introduces recent advancements in this area.

For a pair of coupled chiral particles, the total electromagnetic fields including the initial incident fields and the scattered fields from the other particle can be computed using the coupled-dipole approximation [91,92]. The relevant equations are as follows:

(15) $$\begin{array}{l} E_{o}=E_{\mathrm{inc},o}+\overset{↔}{G_{e}}(d)p_{d}+\frac{1}{c}G_{m}(-d)m_{d},\\ H_{o}=H_{\mathrm{inc},o}+\frac{1}{Z_{b}}[-G_{m}(-d)p_{d}+\frac{1}{c}G_{e}(d)m_{d}],\\ E_{d}=E_{\mathrm{inc},d}+\overset{↔}{G_{e}}(d)p_{o}+\frac{1}{c}G_{m}(d)m_{o},\\ H_{d}=H_{i\mathrm{nc},d}+\frac{1}{Z_{b}}[-G_{m}(d)p_{o}+\frac{1}{c}G_{e}(d)m_{o}], \end{array}$$

with [34,88,93]

(16) $$\begin{array}{l} p_{o}=\alpha_{ee,o}E_{o}+\alpha_{em,o}\mu H_{o},\\ m_{o}=-\alpha_{em,o}E_{o}+\alpha_{mm,o}\mu H_{o},\\ p_{d}=\alpha_{ee,d}E_{d}+\alpha_{em,d}\mu H_{d},\\ m_{d}=-\alpha_{em,d}E_{d}+\alpha_{mm,d}\mu H_{d}, \end{array}$$

where the subscript *o*, *d* correspond to two chiral particles located at *x* axis, with distance *d* between them. The terms

(17) $$\begin{array}{c} \overset{↔}{G_{e}}(d)=\frac{e^{ikd}}{4\pi \varepsilon d^{3}}[(-k^{2}d^{3}-3ikd+3)\frac{d{\left(d\right)}^{T}}{d^{2}}+(k^{2}d^{2}+ikd-1)\overset{↔}{I}]\cdot ,\\ G_{m}(d)=\frac{ik(ikd-1)e^{ikd}}{4\pi \varepsilon d^{3}}d\times , \end{array}$$

refer to electric- and magnetic-field propagators (also known as dyadic Green’s functions). To calculate the optical force on each particle, as defined by Equation (12), one should replace the electromagnetic field and dipole moment in the original expression with those described in Equations (15) and (16) [89].

**Figure 6:**
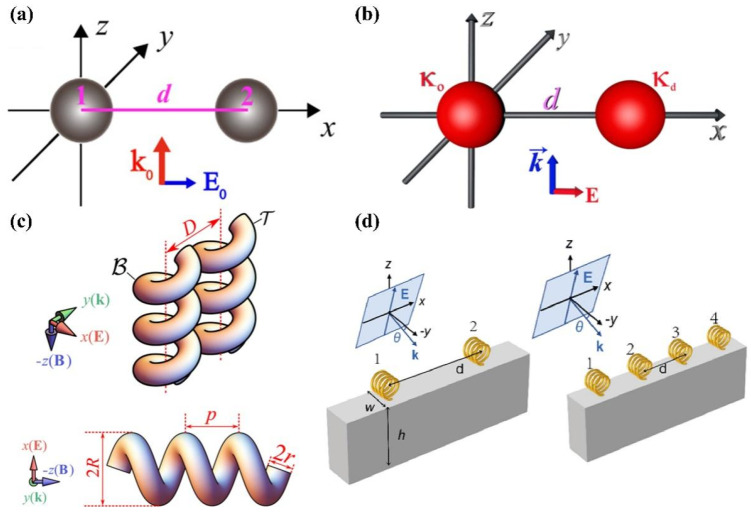
Optical forces on coupled chiral particles. (**a**) Paired spherical particles illuminated by a linearly polarized plane wave traveling in the *z* direction, the distance between two chiral particles equals to *d*, reproduced with permission from ref. [88]. Copyright @ 2015 Optical Society of America. (**b**) Sketch of a pair of spherical particles with the same size and different chirality (one is $\kappa_{o}$ and another is $\kappa_{d}$) illuminated by a linearly polarized plane wave propagating along the *z* direction, reproduced with permission from ref. [39]. Copyright @2020 American Physical Society. (**c**) **Up**: coupled chiral particles formed by two gold helical chiral particles. **Down**: side view of the model system, reproduced with permission from ref. [28]. Copyright @2020 American Physical Society. (**d**) Left: two chiral particles coupled via a silicon waveguide under the incidence of a linearly polarized plane wave. Right: four chiral particles coupled via a silicon waveguide under the incidence of a linearly polarized plane wave, reproduced with permission from ref. [94]. Copyright @2023 Optica Publishing Group.

As illustrated in Figure 6a [88], a chiral dimer, consisting of two chiral nanoparticles with opposite handedness and separated by distance *d*, is illuminated by a linearly polarized plane wave. Chen et al. demonstrated that, when chiral particles with opposite handedness are illuminated, they experience equal optical forces, which causes them to move laterally. Importantly, the direction of the optical force is influenced not only by the distance between the particles but also by their chirality. This finding highlights the intricate interplay between particle separation and chirality in determining optical behavior.

Optical binding refers to the fascinating phenomenon where light-mediated interparticle interactions lead to the self-organization of microscopic particles. For instance, Shi et al. demonstrated that the binding distances between a pair of spherical particles with different chirality, when illuminated by a linearly polarized plane wave [39], vary with the polarization direction, as depicted in Figure 6b. They also observed that significant lateral forces arise due to the particles’ chirality. These forces result not only from the gradient of the optical potential but also from additional factors, highlighting the complex interplay between light polarization and the particle’s chirality in determining binding dynamics.

Through numerical simulations and analytical methods, Kah Jen Wo et al. [28] demonstrated that resonance modes can significantly enhance the interaction forces between coupled chiral particles, with these forces varying depending on the particles’ handedness. The electric dipole force is predominant, although the total force also includes contributions from both electric and magnetic dipoles, as shown in Figure 6c. These findings are crucial for advancing optical manipulation and chiral light–matter interactions, with potential applications in enantiomer selection.

Zhang et al. [94] reported that employing unidirectional coupling of chiral particles positioned on a dielectric waveguide, as illustrated in Figure 6d, can enhance the optical forces on the coupled chiral particles at exceptional points (EPs). They found that with higher-order EPs, the optical force becomes even stronger. Intriguingly, the optical force exhibits a “skin effect”, where the optical force reaches its maximum for the chiral particle located at one end of the lattice. This finding underscores the unique behavior of optical forces in coupled systems, particularly in relation to the position of particles within the lattice.

In summary, the study of optical forces on coupled chiral particles has revealed a complex and rich landscape of interactions that are significantly influenced by the particles’ chirality, separation, and the characteristics of the illuminating light [95,96]. Research has shown that phenomena such as optical binding and unidirectional coupling can cause significant variations in the forces experienced by chiral particles, with factors like polarization direction, exceptional points, and system configuration playing critical roles. The discovery of effects such as the optical force “skin effect” and the dependence of binding distances on chirality and polarization further highlights the intricate interplay between these factors. Future research exploring higher-order EP, more advanced system architectures, and the utilization of advanced materials like metamaterials and plasmonic structures could open up new avenues for controlling and enhancing optical forces in coupled chiral systems. These advancements have the potential to revolutionize applications in optical trapping, enantioselective separation, and the manipulation of nanoscale and mesoscale chiral structures.

## 3. Applications

Recently, the application of optical forces on chiral particles has garnered widespread attention from the scientific community. One of the most prominent applications is the trapping and sorting of chiral particles [26,27,29,35,36,89,97,98,99,100,101,102,103,104,105,106]. This technique provides a non-invasive method for manipulating particles based on their chirality, which is crucial in fields such as chemistry and pharmaceuticals. The ability to selectively control and separate chiral particles has significant implications for improving drug manufacturing processes and advancing material science research, where chirality is a fundamental factor.

**Figure 7:**
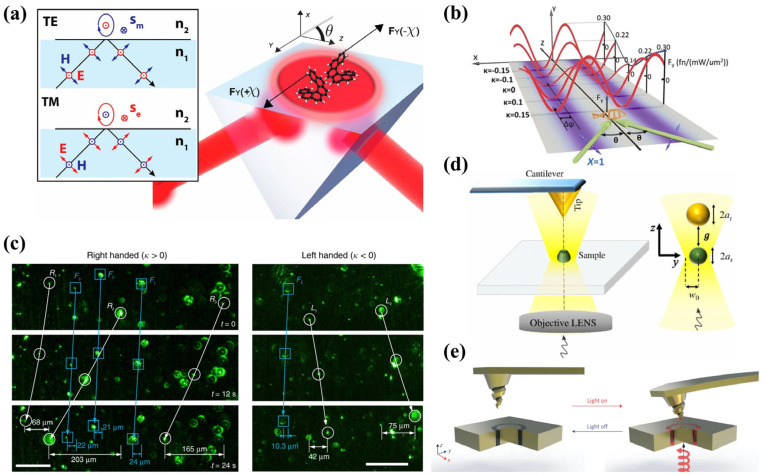
Several applications of optical forces on chiral particles. (**a**) In an evanescent wave field, chiral particles with different chirality experience OLFs in opposite directions. The OLF on a particle is related to its chiral polarizability, reproduced with permission from ref. [27]. Copyright @2015 Proceedings of the National Academy of Sciences. (**b**) In an interference field with mirror-diagonal polarization states, the transverse force on a chiral object always has a sinusoidal form. Its magnitude and direction depend on the magnitude and sign of the chirality, reproduced with permission from ref. [36]. Copyright @2017 American Chemical Society. (**c**) Experimental demonstration of chiral particle sorting in a linear light field. Left: chiral particles with κ>0 experience a leftward OLF. Right: chiral particles with κ<0 experience a rightward transverse force, reproduced with permission from ref. [29]. Copyright @2020 Springer Nature. (**d**) A schematic diagram of an optical force microscope capable of detecting sample chirality based on optical force measurement, reproduced with permission from ref. [107]. Copyright @ 2017 American Physical Society. (**e**) AFM has been employed to measure chiral optical forces, reproduced with permission from ref. [112]. Copyright @2017 Springer Nature.

The principle behind the optical sorting of chiral particles lies in the interaction between light and the inherent asymmetry of these particles. When exposed to specific light patterns, such as tightly focused vector beams, chiral particles experience forces that depend on their chirality. These forces facilitate the separation of particles with different chirality, enabling precise manipulation and sorting. This capability is particularly valuable in applications requiring the selective handling of enantiomers, such as in pharmaceuticals development, where the efficacy and safety of a drug can be chirality-dependent. Several advancements in the field have demonstrated promising results. For instance, in 2015, Amaury Hayat et al. [27] predicted that materials with chiral optical responses in evanescent fields could experience lateral forces, causing particles with opposite helicities to move in different directions. This phenomenon, governed by the chiral polarizability of the particles, enables effective chirality-based sorting. Figure 7a illustrates the OLFs experienced by chiral particles with different helicities in an evanescent field. This finding underscores the potential of evanescent fields for high-precision sorting of chiral particles, with significant implications for enantioselective chemistry and the development of advanced optical devices.

In addition to evanescent waves, Zhang et al. [36] proposed a method for the passive sorting of chiral particles utilizing chirality-dependent LOFs induced by tailored interference fields. This approach involves creating an interference light field by combining a linearly polarized beam with a circularly polarized beam. While the trapping force generated by this configuration is insensitive to chirality, the LOF is highly sensitive to it. As a result, particles with opposite chirality experience different lateral forces, leading to their separation. This method effectively separates chiral particles, as depicted in Figure 7b, and presents a promising technique for precise chiral particle sorting with potential applications in various scientific and industrial fields.

Traditional methods for sorting chiral particles typically focus on larger particles within the geometric optics regime. However, to achieve stable sorting of Mie chiral particles, Shi et al. [29] employed a novel approach by placing polymerized liquid crystal microparticles with different handedness at the air–water interface, as shown in Figure 7c. When these particles were illuminated with line-shaped linearly polarized light beams, bidirectional sorting of chiral microparticles with opposite handedness was observed. This sorting was driven by OLFs, which were influenced by particle size, chirality, angle of incidence, and the polarization of the light beam. This method represents a significant advancement in the precise manipulation of smaller chiral particles and offers a versatile approach for applications in material science and biomedical engineering.

Another important application of optical forces in chiral particles research is chiral detection [107,108,109,110], which uses differential optical forces to analyze non-chiral probes near chiral samples. As illustrated in Figure 7d, Mohammad Kamandi et al. [107] investigated how differential forces on these probes can reveal the enantiomeric composition of chiral samples. Their findings demonstrated that these forces are closely related to the type of enantiomer present, providing a novel method for enantioselective detection. This approach offers a promising avenue for accurately identifying and quantifying enantiomers, which is critical in fields such as pharmaceuticals, where the specific enantiomer of a compound can significantly impact its efficacy and safety.

Quantifying the different magnitudes of optical forces arising from different chirality and detecting changes in these forces can facilitate chiral sorting, enable optical control of enantiomeric chemical synthesis, and provide new avenues for studying chiral materials [111] and electric fields. However, the small magnitude of enantioselective optical forces makes measuring these forces extremely challenging. In 2017, Zhao et al. [112] designed a plasmonic optical tweezer illuminated from below with circularly polarized light and scanned from above using a chiral atomic force microscopy (AFM) probe, as shown in Figure 7e. This technique enables precise control of optical chirality density at the nanoscale and enhances enantioselective optical forces, making the quantification and detection of these forces possible.

The potential relationships between chiral optical forces and molecular chirality have sparked numerous new ideas and approaches in this rapidly evolving field [113,114,115,116]. Beyond sorting and manipulation, optical forces are being employed in chiral sensing [117], enabling the precise detection and differentiation of enantiomers. For example, differential optical forces can analyze the enantiomeric composition of complex mixtures, which is particularly valuable in pharmaceuticals, where the chirality of a drug molecule can significantly influence its therapeutic effects. Furthermore, optical forces are being explored for developing chiral metamaterials with potential applications in advanced photonic devices and for studying chiral quantum systems, which may reveal insights into the interaction between light and chiral quantum states. These developments underscore the far-reaching implications of chiral optical forces across multiple scientific domains.

## 4. Summary and Outlook

Chiral particles interact with light to generate a variety of optical forces, including OGFs, scattering forces, OPFs, OLFs, and forces on coupled chiral particles. The OGF arises from spatial variations in light intensity, drawing particles toward regions of higher or lower intensity depending on their properties. Scattering force results from momentum transfer during light–particle interaction, typically pushing particles along the light’s propagation direction. The OPF, a counterintuitive phenomenon, draws particles back toward the light source rather than pushing them away. The OLF acts perpendicular to the light’s propagation direction and is often influenced by the light’s polarization state. Unique to chiral particles, optical forces emerge from interaction between light and the particle’s structural chirality, leading to effects sensitive to the handedness of both the particle and the incident light. These diverse forces collectively enable precise manipulation, sorting, and sensing of chiral particles, offering new possibilities in fields such as material science and pharmaceutical development [27,29,36,99,107,108,118].

Understanding the optical forces on chiral particles is crucial due to its wide-ranging implications across various scientific and technological fields. In biophysics, for example, manipulating chiral molecules such as proteins and DNA with optical forces could lead to significant breakthroughs in drug delivery systems and molecular diagnostics. In materials science, precise control over chiral nanostructure assembly through light manipulation could lead to the creation of novel materials with unique optical and electronic properties. Moreover, advancements in this area could deepen our understanding of fundamental light–matter interactions, fostering innovations in photonics, such as highly efficient sensors and next-generation communication devices. These diverse applications highlight the critical role of optical forces on chiral particles in advancing progress across multiple domains [11,30,31,89,112,119].

Chiral particles possess unique optical signatures due to their handedness, and leveraging optical forces can significantly enhance the sensitivity and specificity of chiral detection techniques. This capability is especially valuable in the pharmaceutical industry, where the chirality of drug molecules affects their efficacy and safety. Utilizing optical forces to separate and analyze chiral molecules can enhance drug development and quality control processes, leading to safer and more targeted therapeutic outcomes.

Moreover, precise manipulation of chiral particles enables the fabrication of photonic crystals and metamaterials with tailored optical properties [120,121,122,123,124,125,126,127,128]. These advanced materials can be utilized to develop highly efficient sensors, optical filters, and components for communication technologies. The ability to control the spatial arrangement of chiral particles with light opens new possibilities for designing devices with customized responses to electromagnetic waves.

Despite significant progress in exploring optical forces on chiral particles, several challenges remain. A primary issue is the generally small magnitude of these forces, which required the development of strategies to enhance their effectiveness for better manipulation. Additionally, studying optical forces in multiparticle systems is complex due to intricate multibody interactions. The anisotropy and inhomogeneity of chiral particles further complicate the analysis of optical forces and necessitate additional research. Furthermore, experimental exploration in this area is still in its early stages, highlighting the need for more extensive and detailed studies.

Recently, quantum chiral forces have attracted significant attention [129,130,131,132]. Extending the concept of chiral optical forces to quantum fluctuating fields is directly related to Casimir physics [133,134,135]. The chiral Casimir force represents a specific instance of quantum chiral forces, involving quantum vacuum fluctuations between chiral objects [136,137,138]. In contrast, quantum chiral forces may also include interactions with real photons or other chirality-related quantum fields. These phenomena highlight the significance of chirality in quantum interactions, where the symmetry properties of the objects and fields are crucial in determining the nature and magnitude of the forces. Chirality ultimately plays a pivotal role in areas such as quantum optics and Casimir forces, providing a fascinating perspective on the universal characteristics of chirality in the quantum domain.

In conclusion, despite ongoing challenges, the study of optical forces on chiral particles holds great promise for both theoretical and practical advancements. Continuous innovation in theory, experimentation, and interdisciplinary collaboration positions this field for significant breakthroughs that will drive scientific and technological progress. Future applications may include the development of advanced photonic devices, where enhanced optical forces could lead to highly efficient sensors and communication components; precision medicine, where precise optical manipulation could enable targeted drug delivery and personalized treatments; and nanotechnology, where optical forces could facilitate the assembly and control of complex nanostructures for a variety of technological applications. These advancements are expected to deepen our understanding of optical forces and expand their application across diverse scientific and engineering domains.

## Data Availability

Not applicable.
